# Activation of T helper cells in sentinel node predicts poor prognosis in oral squamous cell carcinoma

**DOI:** 10.1038/s41598-020-79273-3

**Published:** 2020-12-18

**Authors:** Åsa Kågedal, Eric Hjalmarsson, Pedro Farrajota Neves da Silva, Krzysztof Piersiala, Susanna Kumlien Georén, Gregori Margolin, Eva Munck-Wikland, Ola Winqvist, Valtteri Häyry, Lars Olaf Cardell

**Affiliations:** 1 Division of ENT Diseases, Department of Clinical Sciences, Intervention and Technology, Karolinska Institutet, Stockholm, Sweden; 2 Department of Otorhinolaryngology, Karolinska University Hospital, Stockholm, Sweden; 3 Department of Pathology and Cytology, Karolinska University Hospital, Stockholm, Sweden; 4 Department of Clinical Immunology and Transfusion Medicine, Karolinska University Hospital, Stockholm, Sweden

**Keywords:** Head and neck cancer, Oral cancer, Immunology, Adaptive immunity, Lymphatic system, Lymphoid tissues, Tumour immunology

## Abstract

Recurrence in oral squamous cell carcinoma (OSCC) significantly reduces overall survival. Improved understanding of the host’s immune status in head and neck cancer may facilitate identification of patients at higher risk of recurrence and improve patients’ selection for ongoing clinical trials assessing the effectiveness of immune checkpoint inhibitors (CPI). We aimed to investigate Sentinel Node-derived T-cells and their impact on survival. We enrolled prospectively 28 OSCC patients treated at Karolinska University Hospital, Stockholm, Sweden with primary tumour excision and elective neck dissection. On top of the standard treatment, the enrolled patients underwent sentinel node procedure. T cells derived from Sentinel nodes, non-sentinel nodes, primary tumour and PBMC were analyzed in flow cytometry. Patients who developed recurrence proved to have significantly lower level of CD4+ CD69+ in their sentinel node (31.38 ± 6.019% vs. 43.44 ± 15.33%, p = 0.0103) and significantly higher level of CD8+ CD HLA-DR+ (38.95 ± 9.479% vs. 24.58 ± 11.36%, p = 0.0116) compared to disease-free individuals. Survival analysis of studied population revealed that patients with low proportion of CD4+ CD69+ had significantly decreased disease-free survival (DFS) of 19.7 months (95% CI 12.6–26.9) compared with 42.6 months (95% CI 40.1–45.1) in those with high CD4+ CD69+ proportion in their Sentinel Nodes (log-rank test, p = 0.033). Our findings demonstrate that characterization of T-cell activation in Sentinel Node serves as a complementary prognostic marker. Flow cytometry of Sentinel Node may be useful in both patients’ surveillance and selection for ongoing CPI clinical trials in head and neck cancer.

## Introduction

Oral squamous cell carcinoma (OSCC), comprising cancers of the lips, oral tongue and upper and lower gum, affected globally over 350,000 individuals and accounted for nearly 180,000 deaths in 2018^1^. Recently, there has been a rising incidence of oral and oropharynx cancers especially among young adults worldwide^2^. In spite of the recent advances in surgical and oncological treatment, a substantial group of OSCC patients does recur, and currently, 5-year relative survival is estimated at 66.2% (based on data from SEER18^3^ 2010–2016, Oral Cavity and Pharynx Cancer, deaths come from U.S. Mortality and includes all races, both sexes). For decades, cancer research has focused on finding pharmaceutical agents bearing the ability to recognize and destroy cancer cells. However, the recent discovery of immune checkpoint inhibitors (CPI) has proven that the human immune system has the capability to fight cancer on its own. As a result, cancer treatment development is now focused on improving the host’s immunological response towards cancer cells^4–6^.

Metastatic spread to regional lymph nodes is one of the strongest factors adversely affecting the overall survival in patients suffering from OSCC^7–9^. The earlier the recurrence is detected, the better treatment alternatives and prognosis can be offered to affected patients. However, in OSCC, there is no clinically applicable and reliable marker stratifying patients into relapse high- and low-risk groups, as it has been established for hematologic malignancies. Thus, introduction of new prognostic markers in OSCC, enabling to select patients who need more rigorous surveillance and treatment is needed. One of the recent examples proposed by Zhou et al.^10^ is the immune prognostic score system based on integrative enumeration and analyses of seven representative types of TILs in immunohistochemistry which proved to effectively predict patient survival.

Little is known about the role and significance of T lymphocytes in tumour draining lymph nodes (TDLN) in OSCC. To the best of our knowledge, T lymphocytes subpopulations in sentinel node, the natural location for the primary immune response against the tumour, have not been investigated yet. Previously, tumour infiltrating T-lymphocytes (TILs) were studied and the majority of reports indicate the presence of TILs as a favourable prognostic factor in head and neck cancer^11–13^. We hypothesized that sentinel node-derived T cells are a more reliable and representative source for evaluation of affected patient’s oncoimmunological status and therefore have potential to provide better insight into their prognosis and choice of treatment. Taking into account our experience in flow cytometry analysis of TDLN^14^, we prospectively gathered a cohort of OSCC patients and analyzed TILs, T-cells activation in primary tumour, sentinel, non-sentinel nodes and PBMC in relation to recurrence status. Influence of the aforementioned variables on survival was studied to identify prognostic markers.

## Methods

### Experimental model and subject details

#### Cohorts of cancer patients

Twenty-eight oral cancer patients were included in this study, 13 women and 15 men; the median age at the time of diagnosis was 64.5 years. All patients had squamous cell carcinomas, the mobile tongue was the most common location (27) and one patient had cancer in the upper gum. They were all treated with primary surgical resection including neck dissection. Tissues were collected during surgery, including primary tumour, blood and lymph nodes including metastatic nodes if available. Sentinel lymph node procedure was performed in 16 patients; total 25 lymph nodes were classified as sentinel nodes. The clinical and pathological data of all patients included in this study are summarized in Table 1.

**Table 1:** Clinical data of enrolled patients.

	Age	Gender	TNM	Recurrence date	Recurrence site	Time to relapse	Time to death	Time without relapse	Number of LN	Number of SN	Tumour
1	59	F	T2N0M0	–		–	–	36	2	1	1
2	46	M	T1N1M0	–		–	–	34	2	1	1
3	64	F	T2N0M0	18/03	Neck	11	22	11	1	2	0
4	74	F	T1N0M0	19/09	Local	33	-	33	1	1	1
5	73	M	T2N0M0	–		–	–	37	2	1	1
6	74	M	T2N0M0	–		–	–	37	2	0	1
7	65	M	T2N0M0	–		–	–	67	2	0	0
8	51	M	T1N0M0	–		–	–	38	0	2	1
9	65	F	T2N0M0	–		–	–	36	1	1	0
10	52	M	T1N0M0	–		–	–	44	2	1	1
11	54	M	T1N2bM0	–		–	–	44	1	0	1
12	68	F	T2N0M0	–		–	–	42	1	2	0
13	43	M	T2N2bM0	–		–	–	56	3	0	1
14	82	F	T4N2bM0	15/08	Distant	5	5	5	2	0	1
15	44	M	T2N0M0	–		–	–	57	2	0	1
16	49	M	T2N0M0	17/10	Local	20	47	20	2	0	1
17	50	M	T1N0M0	–		–	–	56	1	0	1
18	45	F	T1N0M0	–		–	–	56	1	0	1
19	63	M	T2N2bM0	15/06	Neck	8	9	8	1	0	1
20	85	M	T2N0M0	–		–	–	61	2	0	1
21	72	F	T4N0M0	–		–	–	24	1	1	1
22	65	M	T1N0M0	–		–	–	26	1	1	1
23	68	F	T2N0M0	–		–	–	27	1	3	1
24	41	F	T1N0M0	–		–	–	28	2	1	1
25	80	F	T4N0M0	18/08	Local	6	9	6	2	2	1
26	74	M	T3N0M0	–		–	–	24	3	0	1
27	56	F	T1N0M0	–		–	–	20	2	2	1
28	70	F	T1N0M0	19/01	Neck	9	–	9	1	1	1

#### Tissue sampling process

Samples for this study were collected immediately after surgery. Following resection, neck dissection and tumour specimens were kept at 4 °C. Clinically-evident lymph node metastases and non-metastatic lymph nodes from neck region level 1 up to 5 were identified according to their clinical status. Sentinel nodes were marked out intraoperatively. Lymph nodes were divided and one part was separated and collected for the study; the remaining fraction was submitted for routine clinical pathology. Samples were taken from each SLN, as well as a non-sentinel node for comparison. In cases where any SLN was present, one proximal lymph node and one distant lymph node were chosen. Also, a sample from the primary tumour of the tongue was taken from all except in four cases, due to scarce tumour tissue. Collected samples were placed in tissue storage solution (Miltenyi Biotec, Cologne, Germany) and kept at 4 °C.

### Method details

#### Tumour cell isolation

Tumour Dissociation KIT (Miltenyi Biotec #130-100-008) was used to mechanically and enzymatically dissociate surgical specimens. The tissue samples were cut into small pieces and transferred into a gentleMACS C-tube (Miltenyi Biotec #130-096-334), enzymes included in the kit were diluted and pipetted into the C-tube. All specimens were processed with a Gentle MACS Dissociator, (program (h_tumour_01). Enzyme reactions were performed at 37 °C on a MACSmix tube rotator to ensure continuous rotation of the sample. After dissociation, cells were filtered through a 100 µm cell strainer (BD biosciences #352360). A wash steps were performed with DMEM (Gibco #21331020) supplemented with 10% FBS (Thermo Fisher scientific Cat#10270106). Samples were centrifuged at 400×*g* for 5 min. Cells were re-suspended in brilliant stain buffer (BD biosciences #563794) at 40 × 10^6^ cells/ml.

### Strategy for detection of activated T cells

Blood was lysed with ammonium chloride solution for 10 min and washed with PBS (Gibco #2812-019). Single cell suspensions with purified cells from blood and surgical specimens were stained with antibody panels A or B. A complete list of samples and antibody panels are described in Key Resources Table. After staining for 25 min at room temperature, two washing steps were performed with PBS, 400×*g*, for 5 min. Cells were suspended in PBS with 1% paraformaldehyde (HistoLab #02178) and analysed with flow cytometry LSR FORTESSA (BD biosciences). Analysis of the flow cytometry data was performed with FlowJo version 10.1 (LLC, USA).

Cells were first gated based on side scatter (SSC-A) and forward scatter (FSC-A) to exclude debris. Single cells were next selected based on FSC-H versus FCH-A parameters. Cells were then gated on cell population CD3^+^CD4^+^ and CD3^+^CD8^+^ to detect T cells. CD69, CD71, HLA-DR were analysed individually on CD4^+^ and CD8^+^ T cells. FMO control and internal control cells were used to set the gate. Gating strategy for flow cytometry analysis is described in Supplement Fig. S3.

### Flow cytometry analysis of T cell activation

Markers for T cell phenotyping were analysed on a LSR FORTESSA analyser (BD biosciences). At least 1 × 10^4^ events were recorded. Compensations were performed using single staining on anti-mouse IgG and negative control beads (BD bioscience #552843) with FacsDiva software (v8.0.1, BD biosciences, San Jose CA, USA) and according to BD guidelines. The LRS FORTESSA was validated daily to check instrument performance with CS&T beads (BD bioscience #650621).

### Sentinel node procedure

Sentinel node procedure in oral cancer is an innovative approach developed to increase precision of neck metastasis detection. Sentinel node biopsy is not a part of the routine clinical treatment of OSCC patients and is still considered an experimental method in this cancer form. In this study, the procedure was performed as described in paper by Kågedal et al.^15^. In summary, Technetium Tc 99m-labeled colloidal human serum albumin (Nanocoll®, GE-Healthacare) or Lymphoseek®, Tilmanosept,Cardinal Health) were injected around the tumour 16 h before surgery. A SPECT/CT was performed at the morning of the surgery. Indocyanine green (ICG) was injected immediately preoperatively (2 ml of the ICG solution (10 mg)) around the tumour. The intraoperative identification of sentinel node was performed with a near infra-red camera. A handheld gamma probe was also used to confirm localization of the sentinel nodes according to their radioactivity. The sentinel node procedure was under development at our department during this study, therefore the procedure was slightly different in different cases.

### Quantification and statistical analysis

#### Statistical analysis

**Figure 1:**
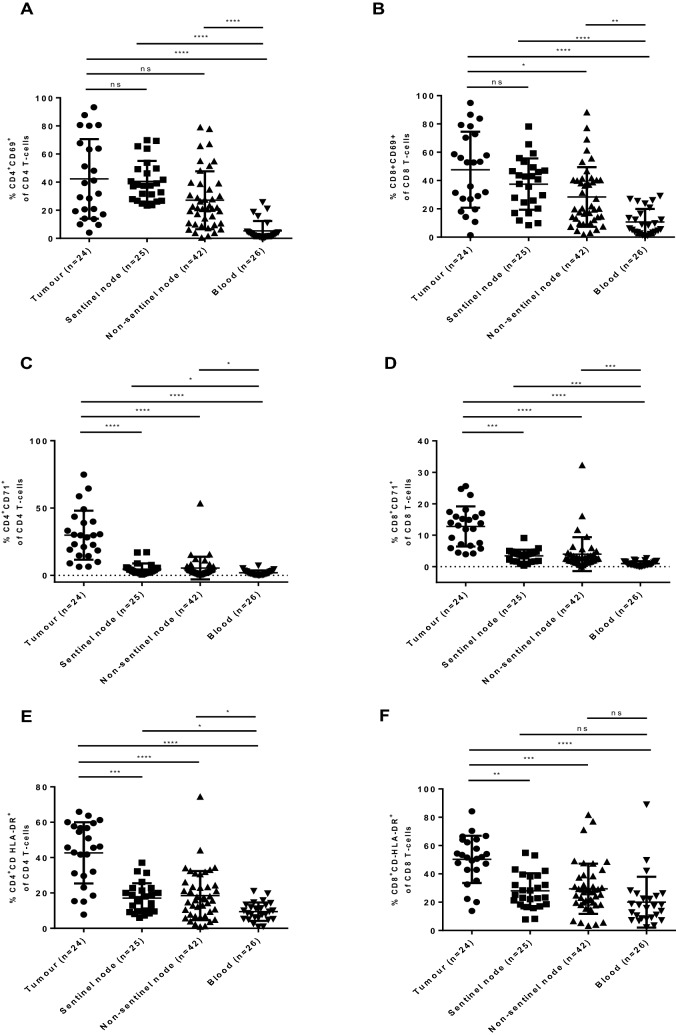
Single cell suspensions from OSCC tumour tissue (n = 24), sentinel node (n = 25), non-sentinel node (n = 24) and PBMC (n = 26) were analyzed by flow cytometry regarding T cell activation surface antigens. Percentages of CD69+ (**A**,**B**), CD71+ (**C**,**D**), HLA-DR + (**E**,**F**) CD4+ and CD8+ differ significantly between analyzed compartments. Mean with SD is represented by solid line and bars within the graph. Kruskal–Wallis test was used to compare groups. *< 0.05, **< 0.01, ***< 0.001, ****< 0.0001.

**Figure 2:**
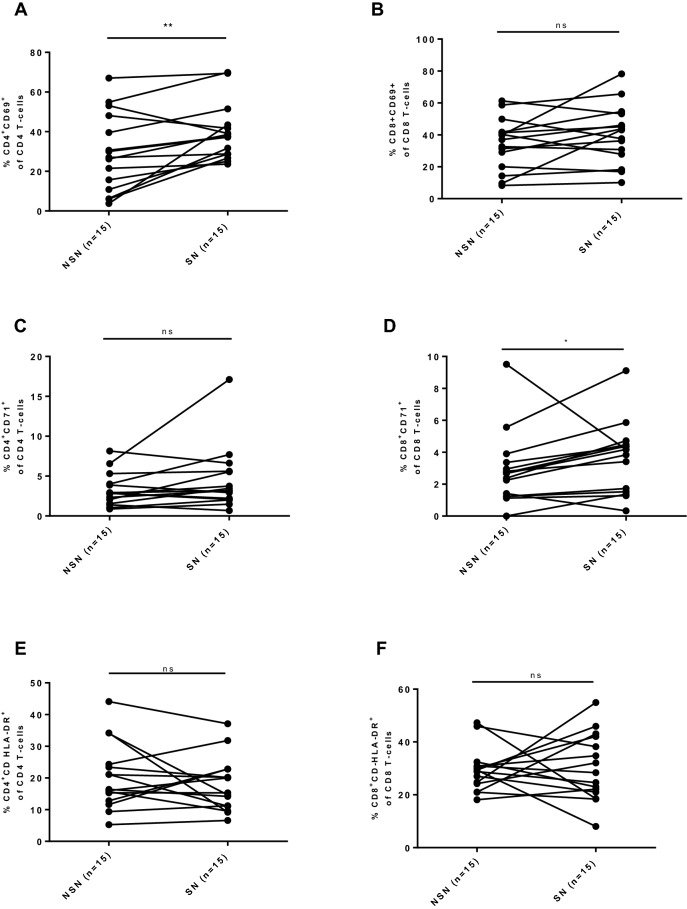
The percentage of activation surface antigens in non-sentinel nodes and sentinel nodes in OSCC patients. All the cases are paired and linked with a line. When more than one sentinel/non-sentinel node was obtained per patient, a mean value was calculated and included into presented analysis. (**A**,**B**,**E**,**F**) compared by paired t-test; (**C**,**D**) compared by Wilcoxon matched-pairs signed rank test. *< 0.05, **< 0.01.

**Figure 3:**
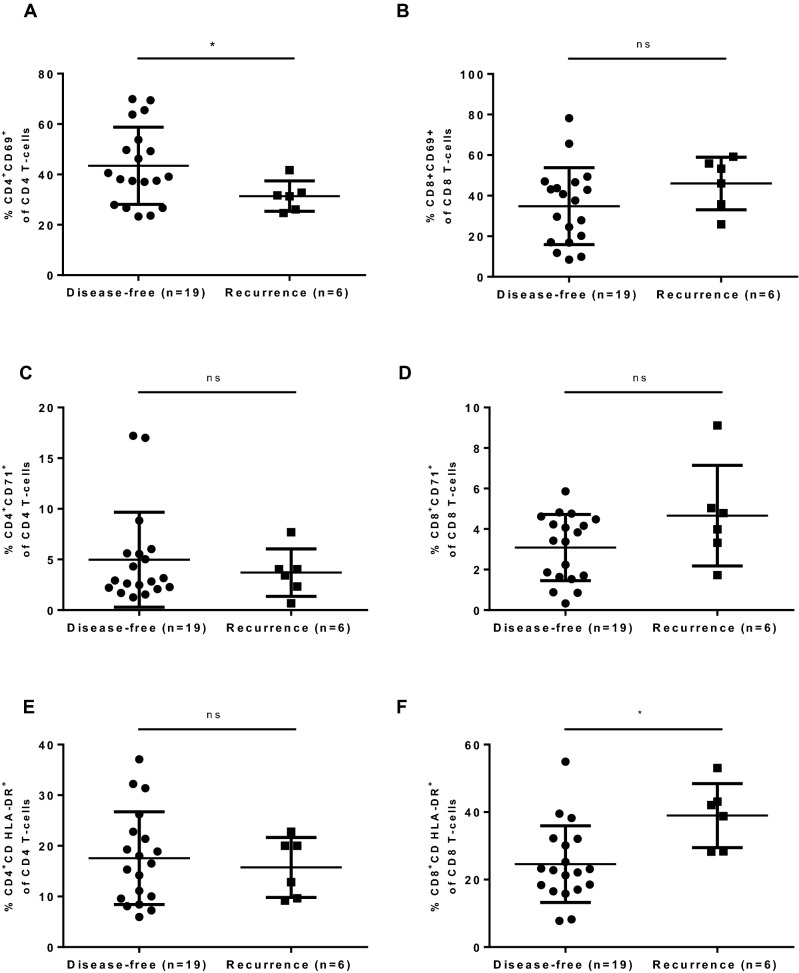
The scatter plot compares the percentage of activation surface markers on CD4+ and CD8+ lymphocytes expressed in sentinel nodes in relation to recurrence status. Sixteen patients contributed with 25 sentinel nodes. Four patients who contributed with 6 sentinel nodes were diagnosed with cancer recurrence during follow-up period. (**A**,**B**,**D**–**F**) were analysed by Unpaired t-test with Welch’s correction. (**C**) was analysed by Mann–Whitney test. *< 0.05.

Statistics: Statistical analyses were performed with GraphPad Prism version 6.01 (GraphPad Software, La Jolla, CA, USA) and IBM SPSS Statistics 26 (IBM Corporation, New York City, NY, United States). The D’Agostino-Pearson normality test was used to determine if data sets were normally distributed for datasets with number of cases over or equal to 10. Kolmogorov–Smirnov normality test was used to determine normality of data sets with number of cases under 10. Based on data normality, Kruskall–Wallis test was chosen to compare different compartments (Fig. 1). In Fig. 2 and Supplementary Fig. S1, paired t test or Wilcoxon matched-pairs signed rank test was used to compare paired groups of data, depending on the distribution of data. In Fig. 3 and Supplementary Fig. S2, unpaired t test with Welch’s correction or Mann Whitney test were used based on distribution of data. Survival analysis was performed by log-rank test comparison and Cox proportional hazard model. The percentage of positive cells for described markers was presented as Mean ± SD. For our binary outcome (recurrence), examining a collection of candidate cut points was reduced to a series of 2 by 2 tables as presented in Supplementary Table S2. The candidate cutpoints were ranked based on a total score obtained from the unadjusted p-value from a chi-square test (highest p-value assigned the lowest score) and corresponding odds ratio estimate (lowest odds ratio estimate assigned the lowest score). The candidate cutoff with the highest score was chosen to be the cutoff in the study. p value < 0.05 was set as significant.

### Ethical approval

The study was approved by the local ethics committee (EPN-Stockholm: 2013/1943–31/4 2015/1650–31/2) and all work was performed in full compliance with the Helsinki Declaration.

### Consent to participate

All patients have signed a written informed consent.

## Results

### Tumour infiltrating T lymphocytes have the highest expression of activation surface markers CD69, CD71 and HLA-DR compared with neck lymph nodes and blood

To investigate T-cell activation in primary tumour, neck sentinel and non-sentinel nodes, we enrolled prospectively 28 OSCC patients treated at Karolinska University Hospital, Stockholm, Sweden with primary tumour excision and elective neck dissection. Clinical data are summarized in Table 1. On top of the standard treatment, the enrolled patients underwent sentinel node identification with use of Tilmanocept tracer or Nanocoll (inclusion and exclusion criteria described in Methods). Single cell suspension from fresh OSCC tumour tissue (n = 24), sentinel nodes (n = 25), non-sentinel nodes (n = 42) and blood (n = 26) was analyzed by flow cytometry to determine expression of activation surface markers on CD4 and CD8 T cells. Immunofluorescence staining for CD69, CD71 and HLA-DR revealed distant activations profiles in the aforementioned compartments.

The percentage of positive cells for all activation markers on both CD4 and CD8 T-cells was highest in primary tumour followed by sentinel node, non-sentinel node and blood. The mean % of positive cells in primary tumour ranged from 12.79 ± 6.376% CD8^+^CD71^+^ to 50.26 ± 16.72% CD8^+^HLA-DR^+^ T-cells, in sentinel node from 3.466 ± 1.939% CD8^+^CD71^+^ to 40.55 ± 14.54% CD4^+^CD69^+^, in non-sentinel node from 3.968 ± 5.401% CD8^+^CD71^+^ to 29.46 ± 17.73% CD8^+^HLA-DR^+^ and in blood from 0.9848 ± 0.7570% CD8^+^CD71^+^ to 20.02 ± 17.97% CD8^+^HLA-DR^+^. Means ± SD for all markers and compartments are summarized in Supplementary Table S1.

### Sentinel node derived T cells express high level of CD69^+^ activation, reflecting activation in primary tumour

When looking into percentage of CD4^+^CD71^+^, CD8^+^CD71^+^, CD4^+^CD HLA-DR^+^ and CD8^+^CD HLA-DR^+^ in different compartments, there was significant difference between primary tumour and sentinel node in expression of aforementioned markers (p-value: < 0.0001, < 0.001, < 0.001 and < 0.01, respectively) (Fig. 1C–F). CD69^+^ activation presented a distinct pattern. There was no significant difference in CD69^+^ marker expression between primary tumour and sentinel node (p-value > 0.05; primary tumour vs. sentinel node: CD4^+^CD69^+^ 42.31 ± 28.34 vs. 40.55 ± 14.54, respectively and 47.66 ± 26.90 vs. 37.48 ± 18.12, respectively) (Fig. 1A,B). However, this association did not persist when analyzed change in mean in 12 individuals contributing both with primary tumour and at least one sentinel node (paired t-test). When paired values were compared, there was significant change in means between sentinel node and primary tumour CD69^+^ activation (p = 0.0314) (Supplementary Fig. S1). When analyzing 15 paired values of mean CD69^+^, CD71^+^, CD HLA-DR^+^ activation in non-sentinel node compared with sentinel node, there was significantly higher representation of CD4^+^CD69^+^ (p = 0.0092) and CD8^+^CD71^+^ (p = 0.0256) in sentinel node compared with non-sentinel nodes (Fig. 2).

### Patients with recurrent OSCC have significantly lower percentage of CD4^+^CD69^+^ and significantly higher of CD8^+^CD HLA-DR^+^ compared with disease-free patients

During the study’s follow-up period seven patients (25.0%) developed recurrence. Four of them (No. 3, 4, 25, 28; Table 1) contributed with sentinel nodes (n = 6). All seven patients contributed with non-sentinel nodes (n = 10).When compared the flow cytometry results stratified by recurrence status, there was a significant difference in expression of CD4^+^CD69^+^ and CD8^+^CD HLA-DR^+^ between groups. Patients who developed recurrence proved to have significantly lower level of CD4^+^CD69^+^ in their sentinel node compared to disease-free individuals (31.38 ± 6.019% vs. 43.44 ± 15.33%, p = 0.0103) and significantly higher level of CD8^+^CD HLA-DR^+^ (38.95 ± 9.479% vs. 24.58 ± 11.36%, p = 0.0116) (Fig. 3). There was no significant difference regarding remaining markers in sentinel node. Levels of activation markers did not correlate with recurrence status when analyzing percentage of studied markers in non-sentinel nodes (Supplementary Fig. S2).

### Patients with low level of CD4^+^CD69^+^ and high level of CD8^+^CD HLA-DR^+^ in sentinel node have significantly worse disease free-survival

**Figure 4:**
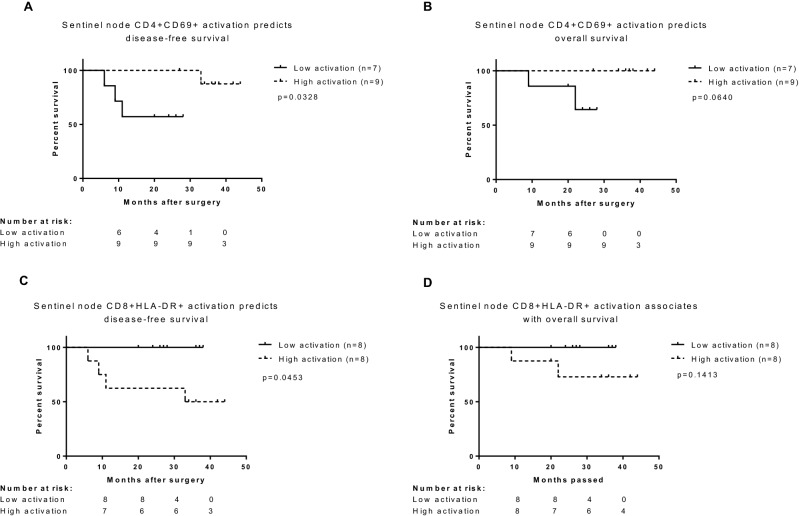
Kaplan–Meier analysis of DFS and OS according to CD4+ CD69+ (**A**,**B**) and CD8 + HLA-DR+ (**C**,**D**) percentage in sentinel nodes. The median value of 37.5% for CD69+ and 25.2% for HLA-DR+ was a priori chosen as the cut-off for separating sentinel nodes with low and high expression of aforementioned surface markers. The p-value for the difference between the two curves was determined by the log-rank test.

The median follow-up for all 28 patients equaled 36 months (range 5–67 months). The median follow-up for 16 patients who contributed with sentinel node equaled 30.5 months (range 9–44 months). Patients’ CD4^+^CD69^+^ and CD8^+^CD HLA-DR^+^ percentage in sentinel node was stratified into low and high expression with cut-off being the median percentage for the marker in sentinel node (37.5% for CD4^+^CD69^+^ and 25.2% for CD8^+^CD HLA-DRIn the studied population, patients with low CD4^+^CD69^+^ had disease-free (DFS) of 19.7 months (95% CI 12.6–26.9) compared with 42.6 months (95% CI 40.1–45.1) in those with high CD4^+^CD69^+^ level (log-rank test, p = 0.033) (Fig. 4A). There was no significant difference in overall survival (OS) (log-rank test, p = 0.0640) (Fig. 4B). On the contrary, patients with low level of CD8^+^CD HLA-DR^+^ had significantly better survival (median months impossible to calculate due to “0” events in group with low activation) (long-rank test, p = 0.0453) (Fig. 4C). Again, there was no significant difference in OS for this marker (long-rank, p = 0.1413) (Fig. 4D). Univariate analysis did not revealed any other factor significantly associated nor with DFS or OS (Table 2). Cox regression analysis did not confirm that level of CD4+ CD69+ and CD8+ CD HLA-DR+ are independent factors influencing DFS or OS (Table 2). The proportional hazards assumption was satisfied for both variables included in the Cox proportional-hazards model (Supplementary Table S2).

**Table 2:** Factors associated with DFS and OS in the studied population.

Factor	No. of patients	No. of relapses	No. of deaths	Mean DFS (95% CI)	DFS (%)	DFS p-value	Mean OS (95% CI)	OS (%)	OS p-value	aOR for relapse (95% CI)	P-value
**Age**											
< 62	6	0	0	–	100.0	0.084	–	100.0	0.246	–	
≥ 62	10	4	2	–	60.0		–	80.0		–	
**Gender**											
Female	11	4	2	–	63.6	0.116	–	81.8	0.300	–	
Male	5	0	0	–	100.0		–	100.0		–	
**pT status**											
T0	8	2	0	37.2 (28.8–45.7)	75.0	0.973	–	100.0	0.173	–	
T +	8	2	2	33.6 (23.5–43.7)	75.0		–	75.0		–	
**pN status**											
N0	15	4	2	–	73.3	0.550	–	86.7	0.699	–	
N +	1	0	0	–	100.0		–	100.0		–	
**Brandwein scale**											
Low risk	1	1	0	33.0 (33.0–33.0)	0.0	0.508	–	100.0	0.660	–	
Intermidiate risk	4	1	0	27.8 (17.1–38.4)	75.0		–	100.0		–	
High risk	10	2	2	36.9 (28.5–42.9)	80.0		–	80.0		–	
**CD4+ CD69+ in sentinel node**											
≤ 37.5 (low)	7	3	2	19.7 (12.6–26.9)	57.1	0.033	–	71.4	0.064	Ref	0.295
> 37.5 (high)	9	1	0	42.6 (40.1–45.1)	88.9		–	100.0		0.004 (0.00–119.70)	
**CD8+ CDHLA-DR+ in sentinel node**											
< 25.2 (low)	8	0	0	–	100.0	0.045	–	100.0	0.141	Ref	0.248
≥ 25.2 (high)	8	4	2	–	50.0		–	75.0		136.5 (0.033–569,208.6)	

Univariate analysis of DFS and OS with p value determined by log-rank test.

Multivariate analysis by Cox proportional-hazards model for factors significant in univariate analysis.

## Discussion

Oncoimmunology is a rapidly expanding research field encouraged by the advent of the new immunotherapies. However, data on the immunology of tumour draining lymph nodes and in particular neck sentinel node is poor. Chen and Mellman^16^ demonstrated the cancer immunity circle where they stressed the important role of the lymph node in cancer immunology and in this project, we proved the clinical significance of this thesis.

The key message of this study is that patients with low proportion of CD4^+^CD69^+^ cells in their sentinel node had significantly decreased DFS of 19.7 months (95% CI 12.6–26.9) compared with 42.6 months (95% CI 40.1–45.1) in those with high CD4^+^CD69^+^ proportion in their sentinel nodes (log-rank test, p = 0.033). On the contrary, those with high proportion in sentinel nodes of CD8^+^CD HLA-DR^+^ presented with significantly worse disease free-survival compared with those who had low level of CD8^+^CD HLA-DR^+^ activation.

These data support the very important observation that T-cell exhaustion or impaired T-cell activation, not only in the tumour but also in the lymph nodes, may play an important role in progression of the disease^17,18^. T helper cells are undeniably the key cell components in the adaptive immunity. Not only are they essential to induce humoral, B-cell dependent immune responses, but they also play a pivotal role in activation of cytotoxic T lymphocytes, which are potentially capable of killing tumour cells. In order to become effector T cell, T helper cells need to be successfully activated by antigen presenting cells (APCs). Tumour cells and tumour microenvironment developed multiple ways to evade immune response against tumours. One of them is inhibiting or impairing T cell activation by expression of PD-L1. In this study, we showed that T helper cells activation is impaired not only in tumour microenvironment but also in the tumour draining lymph nodes, what consequently has a significant impact on survival.

This preliminary data could be a breakthrough in introducing the concept of sentinel node based OSCC immunostaging into clinical practice. The investigation of activated T helper cells may become an important prognostic marker complementary to the TNM classification. We proved that flow cytometry immunoanalysis of sentinel node enables stratification of patients into relapse low- and high-risk group with high efficacy. An improved stratification of patients helps clinicians to identify patients who need a more rigorous follow-up after treatment completion. The next step will be a prospective study enrolling patients into relapse high- and low-risk group based on their T cell activation status in sentinel node. We proved in this report that this method is feasible and may provide valuable prognostic information for patient’s prognosis.

A patient’s immunology status changes constantly over time. T lymphocytes, activated in a tumour draining lymph node, will become tumour-infiltrating lymphocytes (TILs)^19^. The activation markers, included in this study are chosen to reflect the various moments of T-cell activation. CD69 is a marker expressed on recently activated T cells (hours after activation), HLA-DR is expressed later (days–weeks after activation) and CD71, the transferrin receptor, is an intermediate activation marker also expressed on proliferating T-cells^20–22^. Numerous studies indicate that the presence of tumour infiltrating lymphocytes (TILs) is a favourable prognostic factor for therapy outcome and survival in many forms of cancer, including head and neck squamous cell carcinoma^11^. The CD8^+^ T cell subset of TILs and its prognostic value was most frequently assessed in the literature and proven favourable in head and neck cancer^23–25^. However, its role in OSCC remains controversial. The prognostic role of CD4^+^ T cells in tumour microenvironment according to the literature is also ambiguous and still unclear^26^.

In this paper, we demonstrated that it is the sentinel node that harbors important immunological information, which proved to correlate with clinical outcome. On the other hand, investigating lymphocyte fractions in regional lymph nodes (non-sentinel nodes) showed more variability and did not correlate with prognosis. We believe this approach opens an important new perspective for research on both new OSCC prognostic markers in sentinel node. Sentinel node has clear potential to harbor important information that may prove useful in patients’ selection for ongoing CPI clinical trials in head and neck cancer. We hypothesize that patients with low T cell activation in their sentinel nodes are good candidates for immunotherapy. The low T cell activation in tumour draining lymph nodes can reflect a highly immunosuppressive tumour microenvironment and thus, those patients would probably benefit the most from adding the immune checkpoint inhibitors to reinvigorate antitumour immunity.

The idea of lymph nodes being important for cancer patient survival is well established in evidence-based medicine. However, the immunology of lymph nodes in OSCC cancer patients has never been studied the way we present in this study. In the 8th edition of UICC TNM^27^ classification book it is defined how many lymph nodes neck dissection (ND) should include (selective ND—10 or more; radical or modified ND—15 or more). The use of lymph node ratio (LNR), (number of positive nodes over total number of lymph nodes in a neck dissection) has been proven to be a prognostic factor^28^. A high ratio correlated with significantly worse prognosis. Nevertheless, the clinical implementation of this technique has some limitations, mainly due to number of lymph nodes per dissection needed (minimum 18) to achieve significance^29^. In the clinical setting, there are so many variables to evaluate, i.e. when was selective neck dissection chosen or how meticulous was the pathologist in search for LN in the received sample. Therefore, the sentinel node biopsy in OSCC is a promising approach to improve oral cancer management^30^. With our method, 1–4 lymph nodes per patient were included in analysis and provided reliable and consistent results. The advantage of flow cytometry is the cost- and time-effectiveness, as the analysis can be obtained within 3 h after biopsy and the cost per patient does not exceed $200.

Our study has certain limitation, with the greatest being the sample size. The results will need to be confirmed in larger cohorts. Another limitation to this study is a skewed representation of T-stage within the studied group. The group was predominantly represented by patients with small tumours (T1–T2), whereas patients with bigger lesions (T3–T4) were significantly underrepresented. As small tumours may differ immunologically from larger tumours, it is challenging to apply our findings to all TNM stages of OSCC. Furthermore, patients who contributed with sentinel nodes were mostly N0 what also can be a confounding factor in our dataset. Our project is still ongoing and we are constantly enrolling new patients who undergo the same type of investigation as patients included in this report.

## Conclusion

Patients with an increased fraction of activated CD4^+^CD69^+^ and low proportion of CD8^+^CD HLA-DR^+^ T-cells in sentinel node have significantly better disease-free survival. We demonstrate that the use of flow cytometry to characterize activation of T-cells in sentinel node may serve as an important prognostic marker, which could improve patients’ surveillance strategy and overall survival.

## Supplementary Information

Supplementary Information.
