# Supplementary material for: Activation of T helper cells in sentinel node predicts poor prognosis in oral squamous cell carcinoma
Source: Sci Rep. 2020 Dec 18;10:22352. doi: 10.1038/s41598-020-79273-3 (PMC7749121; doi:10.1038/s41598-020-79273-3)
Supplement: Supplementary file 1 — Supplementary Information. [file 41598_2020_79273_MOESM1_ESM.pdf]

SUPPLEMENTARY INFORMATION

Title**: Low proportion of activated $CD4^+$ T helper cells in Sentinel Node predicts worse disease-free survival in oral squamous cell carcinoma.**

Å. Kågedal[1,2] , E. Hjalmarsson[1,2], P. Farrajota Neves da Silva[3], K. Piersiala[1,2], S. Kumlien Georén[1,2], G. Margolin[1,2], E. Munck-Wikland[1,2], O. Winqvist[4], V. Häyry[1,2], L. Olaf Cardell[1,2]

[1]Division of ENT Diseases, Department of Clinical Sciences, Intervention and Technology, Karolinska Institutet, [2]Department of Otorhinolaryngology, Karolinska University Hospital, Stockholm, Sweden.[3]Department of Pathology and Cytology, Karolinska University Hospital, Stockholm, Sweden.[4]Department of Clinical Immunology and Transfusion medicine, Karolinska University Hospital, Stockholm, Sweden.

Key Resources Table		
**REAGENT or RESOURCE**	**SOURCE**	**IDENTIFIER**
Antibodies		
anti-CD3 V450	BD Biosciences	Cat#560365 AB_1645570
anti-CD4-PerCP/Cy5.5	BD Biosciences	Cat#560650 AB_1727476
anti-CD8 BV510	BD Biosciences	Cat#563919 AB_2722546
anti-CD69-FITC	BD Biosciences	Cat#555530 AB_395915
anti-CD69-PE	BD Biosciences	Cat#555531 AB_395916
anti-CD71-APC	BD Biosciences	Cat#551374 AB_398500
anti-CD154 PE-CF594	BD Biosciences	Cat#555701 AB_396051
anti-CD227 FITC	BD Biosciences	Cat#560651 AB_1727528
anti-HLA-DR-PE-CY7	BD Biosciences	Cat#560651 AB_1727528
anti-PD-1 PE	BD Biosciences	Cat#560795 AB_2033989
CK5/8 FITC	Novus Biologicals	Cat#NBP1-97730
EpCam FITC	BD Biosciences	Cat#347197 AB_400261
DAPI	Thermo Fisher scientific	Cat#D1306
Tissues		
Fresh tumour	Karolinska University Hospital	N/A
Fresh lymph node	Karolinska University Hospital	N/A
Fresh blood	Karolinska University Hospital	N/A
Chemicals		
NH4CL	Merk	Cat#A412945321
NAHCO3	Merk	Cat#S5761
DisodiumEDTA	Sigma	Cat#E9884
Formaldehyd solution 4%	HistoLab	Cat#02178
Mowiol	Sigma	Cat#324590
DABACO	Sigma	Cat#290734
Glycerol	Sigma	Cat#G5516
Critical Commercial Assays		
PerFix-nc	Beckman Coulter	Cat#B10825
Tumour Dissosiation, Human	Miltenyi Biotec	Cat#130-095-929
Software and Algorithms		
Graphpad	Graphpad	N/A
Flowjo	BD Biosciences	N/A
BD FacsDiva	BD Biosciences	N/A
Other		
DMEM	Gibco	Cat#21331020
MACS Tissue Storage	Miltenyi Biotec	Cat#130-100-008
Gentle Macs C tubes	Miltenyi Biotec	Cat#130-096-334
FC-Block	BD Biosciences	Cat#564220
PBS	Gibco	Cat#2812-019
Briliant stain buffer	BD Biosciences	Cat#563794
Compbeads	BD Biosciences	Cat#552843
CS&T beads	BD Biosciences	Cat#650621
6 Peak Validation Beads	BD Biosciences	Cat#653145
Cellstrainer (100µm)	BD Biosciences	Cat#352360
BD Vacutainer	BD Biosciences	Cat#367526
FBS	Thermo Fisher scientific	Cat#10270106

Key Resources Table. Contact for Reagent and Resource Sharing. Further information and requests for resources should be directed to, and will be fulfilled by the Lead Contact, Lars Olaf Cardell lars-olaf.cardell@ki.se).

Supplementary Figure 1. The percentage of activation surface antigens in sentinel nodes and primary tumours of OSCC patients. All the cases are paired and linked with a line. When more than one sentinel node was obtained per patient, a mean value was calculated and included into presented analysis. A, B, D, E, F compared by paired t-test; C compared by Wilcoxon matched-pairs signed rank test. *<0.05, **<0.01, *** <0.001, ****<0.0001

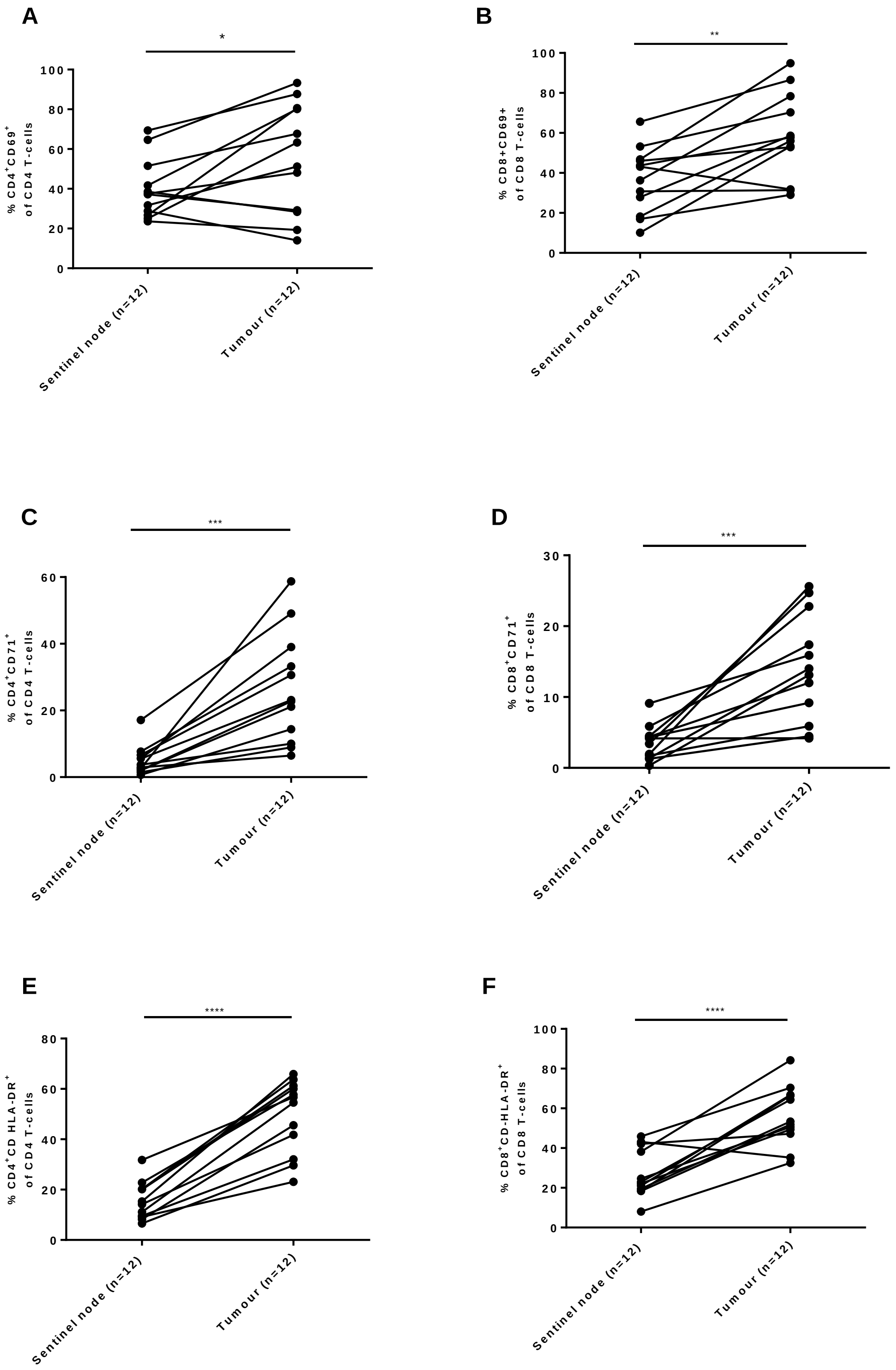

Supplementary figure 2. The scatter plot compares the percentage of activation surface markers on CD4+ and CD8+ lymphocytes expressed in non-sentinel nodes of OSCC patients in relation to recurrence status. Twenty-eight patients contributed with 44 non-sentinel nodes. Six patients who contributed with 8 sentinel nodes were diagnosed with cancer recurrence during follow-up period. A,B,D,E,F were analysed by Unpaired t-test with Welch's correction. C was analysed by Mann-Whitney test. *<0.05

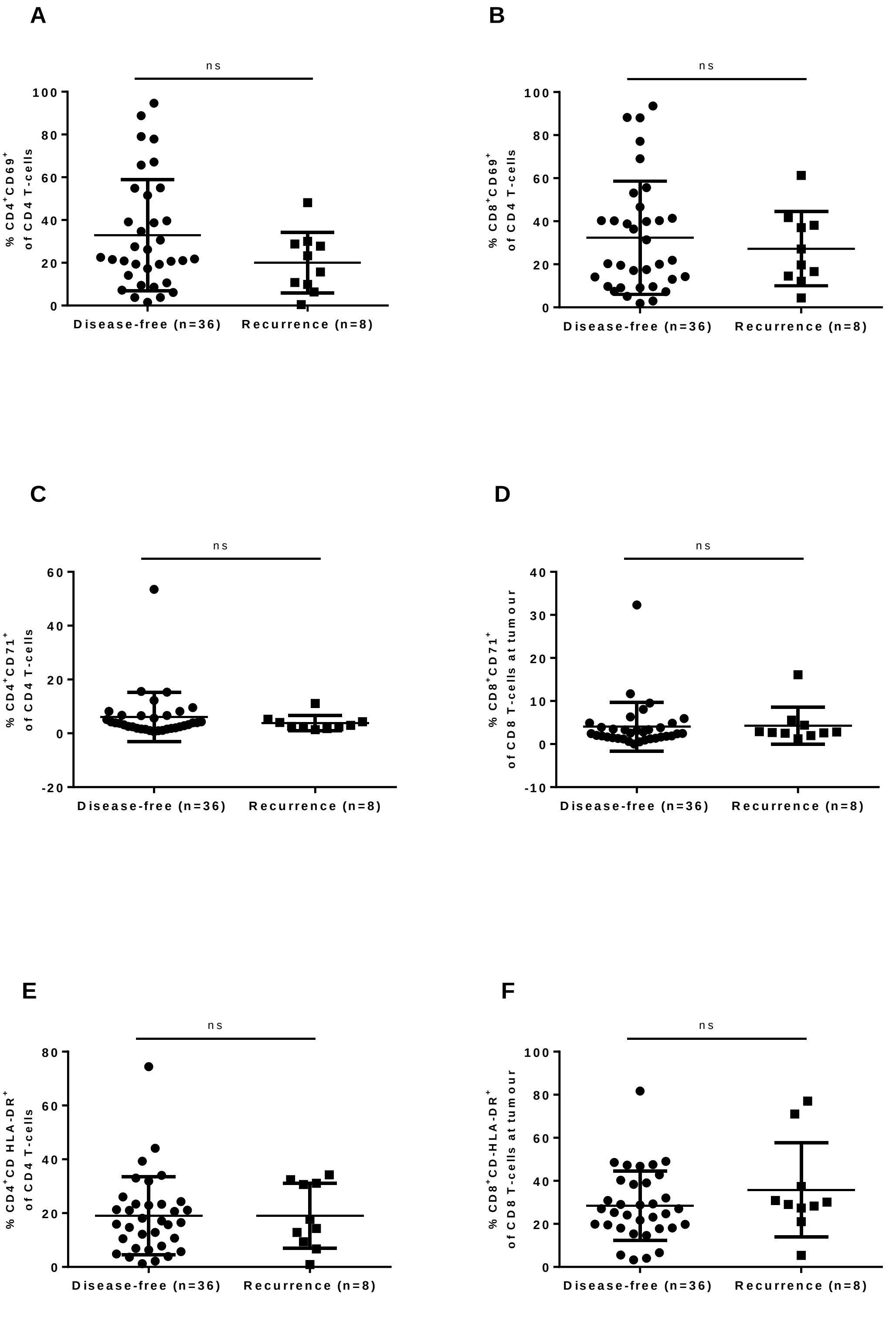

Supplement Figure 3. *Detection of activation markers expressed on CD4⁺ T cells in lymph nodes*. FMO control and internal control cells were used to set the gate. CD69 expression is upregulated on T cells only hours after stimulation, and the expression diminish after 2-3 days. CD71 is also upregulated on T cells around 12 h after stimulation and HLA-DR is upregulated >24 hours after stimulation on differentiating and proliferating T cells.

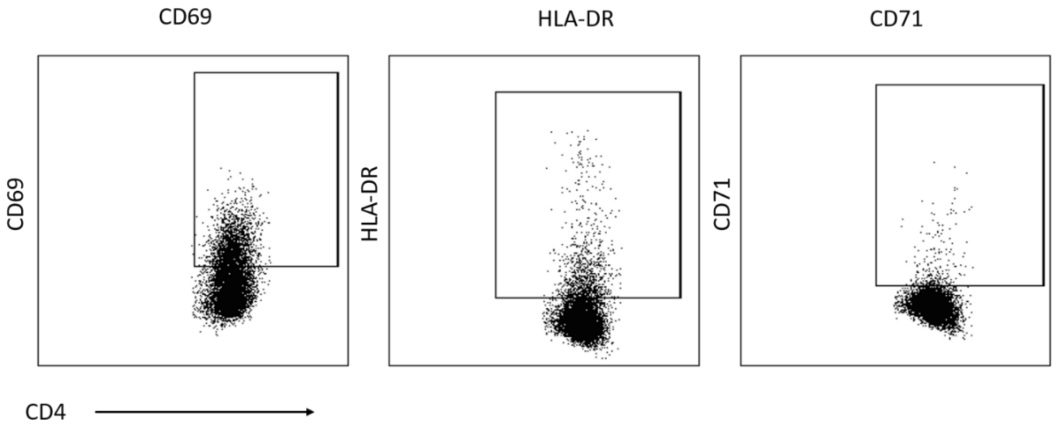

Supplementary Table 1. Mean ± SD of the percentage CD69$^+$, CD71$^+$ and CD HLA-DR$^+$ CD4$^+$ and CD8$^+$ T lymphocytes in primary tumor, sentinel nodes, non-sentinel nodes and blood of OSCC patients.

Compartment	CD4$^+$CD69$^+$	CD4$^+$CD71$^+$	CD4$^+$CD HLA-DR$^+$	CD8$^+$CD69$^+$	CD8$^+$CD71$^+$	CD8$^+$CD HLA-DR$^+$
Primary tumor	42,31 ± 28,34	29,87 ± 18,21	42,64 ± 17,29	47,66 ± 26,90	12,79 ± 6,376	50,26 ± 16,72
Sentinel node	40,55 ± 14,54	4,671 ± 4,231	17,13 ± 8,401	37,48 ± 18,12	3,466 ± 1,939	28,03 ± 12,44
Non-sentinel node	27,10 ± 20,60	5,476 ± 8,396	18,49 ± 13,87	28,35 ± 21,09	3,968 ± 5,401	29,46 ± 17,73
PBMC	5,285 ± 7,030	1,940 ± 1,805	9,439 ± 5,187	10,71 ± 9,227	0,9848 ± 0,7570	20,02 ± 17,97

Supplementary table 2. (A) Report of the assumption of proportional hazard for variables included in Cox Regression Model. (B) Estimation of optimal cut points.

A.

Cox regression with a time-dependent covariate for “CD4+CD69+ in sentinel node > median variable”

	B	SE	Wald	df	Sig.	Exp(B)	95,0% CI for Exp(B)	
							Lower	Upper
OverSNMedianCD4CD69	-3.870	20.955	.034	1	.853	.021	.000	14328721290333778.000
T_COV_	-.094	2.382	.002	1	.968	.910	.009	96.944

Cox regression with a time-dependent covariate for “CD8+ CD HLA-DR+ in sentinel node > median variable”

	B	SE	Wald	df	Sig.	Exp(B)	95,0% CI for Exp(B)	
							Lower	Upper
OverSNMedianCD8CDHLA	4.508	7.195	.393	1	.531	90.743	.000	120956670.884
T_COV_	-.021	.409	.003	1	.959	.979	.439	2.182

B.

For our binary outcome (recurrence), examining a collection of candidate cutpoints was reduced to a series of 2 by 2 tables as presented under. The candidate cutpoints were ranked based on a total score obtained from the unadjusted p-value from a chi-square test (highest p-value assigned the lowest score) and corresponding odds ratio estimate (lowest odds ratio estimate assigned the lowest score). The candidate cut-off with the highest score was chosen to be the cutoff in the study.

	x ≤ Median	x > Median
Recurrence (n)	3	1
Non-recurrence (n)	4	8
	x ≤ Mean	x > Mean
Recurrence (n)	3	1
Non-recurrence (n)	7	5
	x ≤ 50%	x > 50%
Recurrence (n)	4	0
Non-recurrence (n)	12	4

Candidate cut-off	Total score	Odds ratio (score)	Unadjusted p-value (score)
Median	6	10.67 (3)	0.1058 (3)
50%	4	3.240 (2)	0.5377 (2)
Mean	2	2.143 (1)	1.0000 (1)
